# Analysis of computational approaches for motif discovery

**DOI:** 10.1186/1748-7188-1-8

**Published:** 2006-05-19

**Authors:** Nan Li, Martin Tompa

**Affiliations:** 1 Department of Computer Science and Engineering, Box 352350, University of Washington, Seattle, WA 98195-2350, USA

## Abstract

Recently, we performed an assessment of 13 popular computational tools for discovery of transcription factor binding sites (M. Tompa, N. Li, et al., "Assessing Computational Tools for the Discovery of Transcription Factor Binding Sites", Nature Biotechnology, Jan. 2005). This paper contains follow-up analysis of the assessment results, and raises and discusses some important issues concerning the state of the art in motif discovery methods: 1. We categorize the objective functions used by existing tools, and design experiments to evaluate whether any of these objective functions is the right one to optimize. 2. We examine various features of the data sets that were used in the assessment, such as sequence length and motif degeneracy, and identify which features make data sets hard for current motif discovery tools. 3. We identify an important feature that has not yet been used by existing tools and propose a new objective function that incorporates this feature.

## 

For the past decade, research on identifying regulatory elements, notably the binding sites for transcription factors, has been very intense. The problem, usually abstracted as a search problem, takes as the input a set of sequences, which encode the regulatory regions of genes that are putatively co-regulated. The output consists of the regulatory elements (short words in the input sequences) and a motif model that profiles them.

Numerous computational tools have been developed for this task. Natually, evaluation of these tools is becoming vital in this area. Recently, Tompa et al. [1] report the results of one such assessment. In this assessment, some popular tools are tested on datasets of four species: human, mouse, fly and yeast. Each dataset contains a set of sequences planted with binding sites of one transcription factor. The binding sites are provided in the TRANSFAC database [2]. Details of the datasets are explained in [1].

Besides the result of the assessment, this work also raises questions about the approaches used by these tools. We discuss some interesting questions that arise from further analysis of the assessment in [1]. We believe that techniques that have been adopted in search are very powerful, as proven by these eminent tools. But the definition of the search problem, especially the formulation of objective functions, leaves space for substantial improvement in the performance of the motif discovery tool.

## 1 Are the objective functions informative?

The first step to design a new algorithm for the motif discovery problem is to choose a proper objective function. This is critical because the objective function implements the designer's understanding of the protein-DNA interaction model. Searching for candidates that optimize the objective function is a major step to pull out the candidate binding sites from the background sequences. An ideal objective function should be able to assign the optimal score to the true motif binding sites and nowhere else.

Although there are numerous tools available, surprisingly the types of objective functions are not as many. Here we examined three popular objective functions. Theoretically, for each objective function we would test whether the score of the planted binding sites is superior to the scores of all other sets of words in the background sequences which are false positive predictions. This, of course, is impractical. In practice, we chose one tool that applies this objective function and compared the tool's prediction, which unfortunately is often a false positive, with the planted motif. If the planted motif has a better score, then the gap between the two scores shows the least extent to which the tool misses the global optimum of the objective function. On the other hand, if the prediction scores higher, it would suggest that the objective function is not accurate enough to model the true binding sites.

### Log likelihood ratio

This ratio and its associated forms are used by most alignment-driven algorithms to assess the significance of motif candidates. When the candidates are of different lengths, the *p*-value of the ratio is used. A method to compute the *p*-value is described in [3]. The log likelihood ratio of the predicted motif *m *is



where *X *is the set of sequences in the dataset, *Pr*(*X*|*φ*, *Z*) is the likelihood of the sequences *X *given the motif model *φ *and its binding sites *Z*, and *Pr(X|p*_0_) gives the likelihood of the sequences assuming the background model *p*_0_.

**Figure 1:**
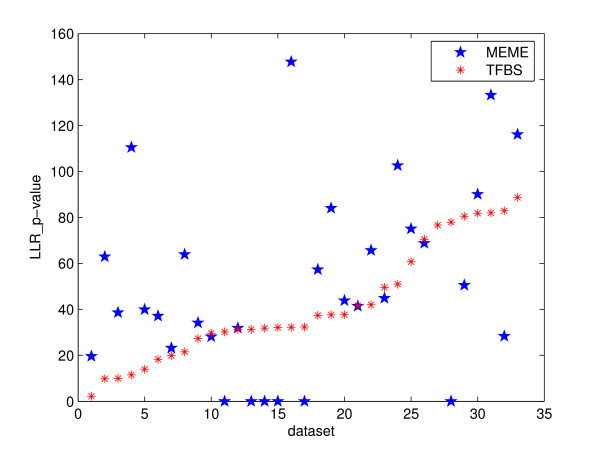
Objective function: *p-*Value of log likelihood ratio in negative logarithm scale. The figure exhibits the comparison of the p-value of the log likelihood ratio between the planted motifs ("TFBS" in the legend) and that of MEME's predictions for selected datasets from [1]: we use only "Generic" and "Markov" among the three types of datasets (see [1]), because in "Real" type datasets the predictions are possibly genuine binding sites of some unannotated transcription factor other than the ones planted. The datasets are sorted in ascending order of TFBS scores for clarity. For each dataset, there are two scores: the score of TFBS and the score of MEME's prediction. Points on the x-axis correspond to the datasets for which MEME didn't make any prediction.

MEME [4] carries out an EM-based procedure to search for a model  that maximizes the likelihood ratio. The local optimum can sometimes be avoided by rerunning the program with different initializations. Figure 1 depicts, for each dataset from [1], the scores (the *p-*values of the log likelihood ratio in the negative logarithm scale) of MEME's predictions and the planted binding sites. For most datasets, the predictions of MEME have higher scores than the planted motifs. We conclude that even an algorithm guaranteeing the global optimal solution for the log likelihood ratio function will miss the true binding sites in these datasets, because this objective function does not accurately capture the nature of the binding sites.

Now, consider one dataset in detail. The dataset is an example for which the planted motif has a higher log likelihood ratio score than MEME's prediction, yet we argue that log likelihood ratio still doesn't work well as an objective function in this case.

In a way, the motif-searching problem is a classification problem: all the words of a certain length appearing in the sequences should be partitioned into two classes: the binding sites, and all the others. Training the optimal classifier equates to searching for the optimal candidate motif model. When the log likelihood ratio is applied as the objective function, the ultimate classifier would be a threshold of the log likelihood ratio score so that all the binding sites are above the threshold, and all the others are below it. A classifier corresponding to a good prediction can achieve a decent balance between the false positives and false negatives of the classification. Vice versa, if no threshold is satisfactory enough to classify the words, no good prediction can be found under this motif model.

**Figure 2:**
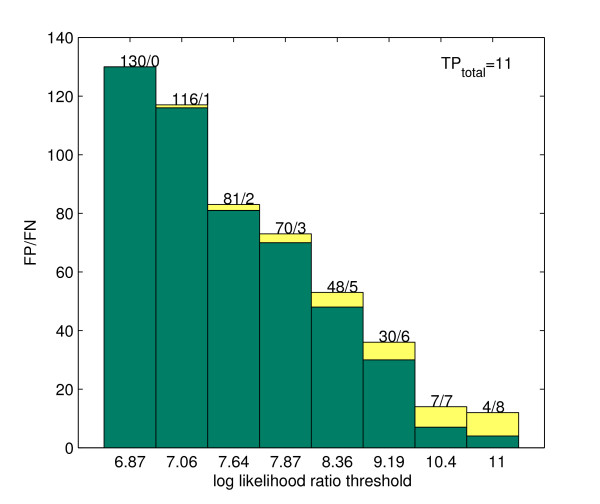
Classifiability using log likelihood ratios as thresholds. Each bar stands for a value of the cut-off threshold to distinguish the binding sites of the motif from background. The pair of numbers on the top of each bar indicate the number of false positives(FP) and the number of false negatives(FN) resulting from the classification.

To test the classifiability of this dataset, we calculated the log likelihood ratio scores of all the words in it, including the true binding sites, and tried out various threshold values to classify the words. Among those having scores above the threshold, the numbers of words are counted which belong to binding sites and which belong to the background sequences. Figure 2 indicates that no matter what threshold we choose to identify the binding sites of the motif, we won't be able to find a value to achieve an acceptable balance between the sensitivity and the specificity of the classification. For example, to correctly classify all 11 true binding sites, the threshold must be chosen so low that 130 false positives are classified as binding sites of the motif.

It is therefore fair to say that log likelihood ratio alone will not be able to separate the true motif from the background noise. We will return to it later.

### Z-score

The Z-score measures the significance of predictions based on their over-representation. YMF [5] searches a restricted space defined by a consensus motif model and finds the candidates with the top Z-scores. The form of the Z-score is as follows:



where *obs*(*m*) is the actual number of occurrences of the motif *m, E*(*m*) is the expected number of its occurrences in the background model, and *σ*(*m*) is the standard deviation.

**Figure 3:**
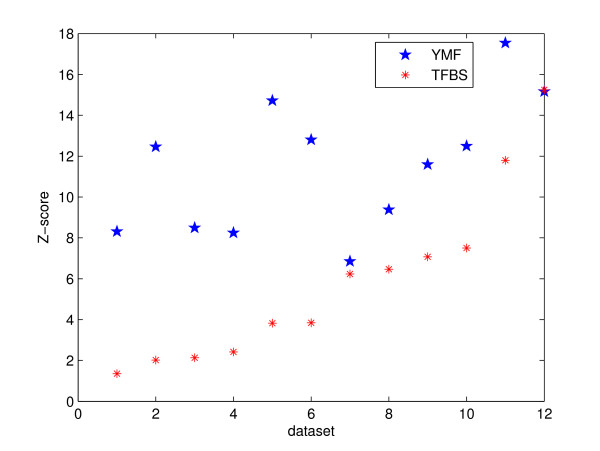
Objective function: Z-score. The figure shows the comparison between the Z-scores of the planted motifs (TFBS in the legend) and the predictions of YMF for some datasets. For the sake of comparison simplicity, we only used datasets ("Generic" and "Markov" types only, for the same reason as in Figure 1) when predictions of YMF and the planted motifs have the same length.

Consensus-based algorithms such as YMF are sometimes criticized for not being able to incorporate the true binding sites into the motif model. To focus on the objective function and spare the limitation induced by the consensus motif model, we fantasize a motif model for each dataset that comprises the planted binding sites completely and exclusively. We calculate the Z-scores of the predictions and the planted motifs for selected datasets, as shown in Figure 3. Note that the competition is actually not fair: with an expanded motif search space, the new optimum should be at least as high as the current prediction. Nevertheless, we consider the Z-score of the prediction as a touchstone: any score lower than it will not be competitive in the new search space. From Figure 3, we see that is exactly what happens in nearly all of the tested datasets. Note the similarity to results as shown in Figure 1 in the sense of our test: statistical over-representation as measured by Z-score does not necessarily mean binding preference either.

### Sequence specificity

Another type of objective function emphasizes the likelihood that most, if not all, sequences are potentially bound by the transcription factor. That means a prediction having multiple binding sites in one sequence and none in the others is much less significant than a prediction having a balanced number of binding sites in each sequence. This idea is designed into ANN-Spec [6] and Weeder [7]. The objective function, named sequence specificity, is defined in [7] as follows.



where *E*_*i*_(*m|p*_0_) is the expected number of motif *m*'s occurrences in sequence *i *assuming the background model *p*_0_, and *L *is the total number of sequences in the dataset.

**Figure 4:**
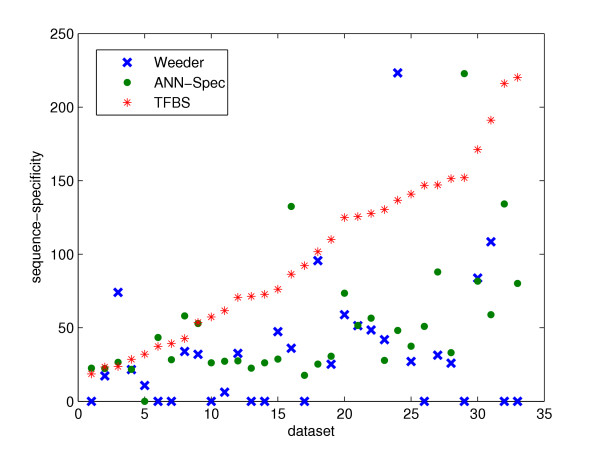
Objective function: sequence specificity score. The figure shows the comparison between the sequence specificity scores of the planted motifs (named TFBS in the legend) and the predictions of Weeder and ANN-Spec. For the same reason as in Figure 1, only datasets of "Generic" and "Markov" types are tested. The x-axis tells the indices of the datasets. The datasets are sorted in ascending order of TFBS scores for clarity. For each dataset, there are three scores: the score of TFBS motif, Weeder's prediction, and ANN-Spec's prediction, colored in red, blue and green respectively. Points on the x-axis corresponds to the datasets for which the tool didn't make any prediction.

We calculated the scores of the predictions of Weeder and ANN-Spec and the planted motifs. The planted motif has a higher score than the predictions of the tools for most datasets, as illustrated in Figure 4. The obvious gap between the scores of planted binding sites and the predictions reflects a lack of optimum of the search strategies adopted by these tools. Recall that ANN-Spec is a generalized version of SEM (Stochastic EM), and Weeder uses a greedy and heuristic search method.

Comparing Figure 4 with the other objective functions (Figure 1, 3), this result shows certain promise that using the sequence specificity score may often lead to the true binding sites. From objective function point of view solely, sequence specificity seems to have the edge for our datasets. An assumption of this objective function is that most sequences in the datasets should have binding sites of the motif. Although our data shows that tools such as Weeder and ANN-Spec are not too sensitive to the slight departure from this assumption, we have not tested them on datasets with more deviation. The Z-score function is based on the statistical over-representation solely without any reference to biological theories. The log likelihood ratio relies on high-quality non-gapped alignments, but it's not clear that non-gapped alignments are powerful enough to model the true binding sites. No objective function meets our standard that all planted motifs should have scores at least as high as those of the predictions. We need to understand better the conservation information hidden among those binding sites.

## 2 Is this a hard dataset?

Among the questions arising from the assessment project, a particularly interesting one is this: what makes a particular dataset so hard to solve? The answer to this question would be helpful at both ends of the tools. For the users, it would save time and money if a certain assurance of the predictions is provided; for the designers, focus would be put upon factors that account for some for the poor performance of current methods.

Some features of the datasets obviously show correlations with the tools' performance. For instance, a dataset of a large size intuitively is not easy to handle. But, when any feature is studied alone, its correlation with the performance of the tools is always too weak to be convincing, as the effects of all but this feature are ignored.

We applied multiple linear regression [8], a method of estimating the conditional expected value of one variable *Y *(the dependent variable) in terms of a set of other variables *X *(predictor variables). It is a special type of regression in which the dependent variable is a linear function of the "missing data" (regression coefficients *W*) in the model. A general form of multiple regression can be expressed as

*E*(*Y*|*X*) = *f*(*W*, *X*) + *ε*

where *f *is a linear function of *W*, a simple example of which is *f*(*W*, *X*) = *W*·*X*. *ε *is called regression residue. It has the expected value 0, and is independent of *X *("inequality of variance").

The goodness-of-fit of regression is measured by the coefficient of determination *R*^2^. This is the proportion of the total variation in *Y *that can be explained or accounted for by the variation in the predictor variables {*X*}. The higher the value of *R*^2^, the better the model fits the data. Often *R*^2 ^is adjusted for the bias brought by the degree of freedom of the model and the limited number of observations as *= R*^2^*- p *× , where *n *is the number of observations, and *p *is the number of predictors.

In our application of multiple linear regression, *Y *is the performance of the tools for a dataset, which is measured by the highest nucleotide-level correlation coefficient score *nCC *(see [9]) among all the tools. The reason for using the highest score is to smooth the disadvantages of each individual tool. The predictor variables are a set of features of a dataset which we think may be possible factors. These features include:

1. the total size of a dataset;

2. the median length of a single sequence in a dataset;

3. the number of binding sites in a dataset;

4. the density of the binding sites, which equals the number of binding sites divided by the total size of a dataset;

5. the fraction of null sequences (ones that do not contain a binding site) in a dataset;

6. relative entropy of binding sites in a dataset;

7. the relative entropy-density in a dataset, which is the overall relative entropy times the density of the binding sites;

8. the uniformity of the binding site locations within the sequences in a dataset. We quantified this position distribution information by performing a Kolmogorov-Smirnov test [10] against a uniform distribution and calculating its *p-*value.

We used least square fitting to calculate the regression coefficients. The most common forms of it include least square fitting of lines and least square fitting of polynomials. In the former, only the first-order term of the predictor variables are involved in the regression model; in the latter, higher order polynomial terms of them are also used. Due to a limited number of observations available (the number of "Generic" and "Markov" datasets in the analysis is about thirty) compared to the number of features, we confined ourselves to the simplest form of linear regression: only the first-order terms are used in the fitting. As we will discuss below, this simplification does not affect the regression result much.

Some features are obviously not independent. For example, relative entropy-density is the non-linear operation (multiplication) of two other *X *variables, relative entropy and density. For every set of features that are highly correlated to each other, we replaced it by its subset with the highest adjusted correlation coefficient .

**Figure 5:**
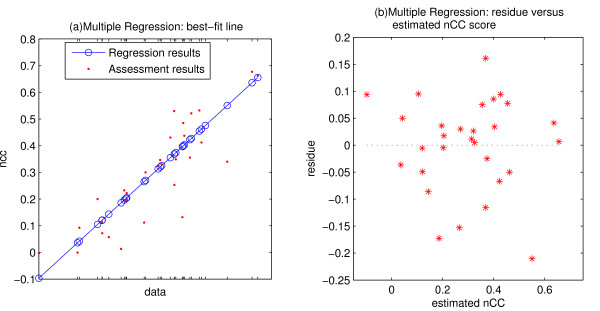
Multiple linear regression result. (a)The best-fit line. Marks on the *x*-axis index the datasets, which are arrayed so that the estimated values of the dependent variable (the assessment scores) are in a straight line. For each dataset, the red dot is the assessment score, measured by the best correlation coefficient score *nCC *(see [9]) among all the tools, and the circle on the blue line shows the estimated value of the best-fit linear model. (b)Residues of the regression versus estimated nCC score. The *x*-axis is the estimated value of the dependent variable, the *y*-axis is the corresponding residue. This plot shows little indication of inequality of variance, which is an important assumption of linear regression.

Then the best subset of features is chosen to maximize the multiple linear regression output. The set of features that shows the most correlation to the performance consists of the relative entropy of the binding site alignment, the position distribution of the binding sites in the sequences, and the length of the single sequence in the dataset. The result is exhibited in Figure 5(a). The adjusted coefficient of determination  is about 68%, with a *p*-value less than 0.001. The regression residues versus the estimated response (Figure 5(b)) doesn't indicate evident inequality of variance, which is an important assumption of linear regression the requires that regression residues are independent of the expected value of *Y*.

We then ran a least square fitting of second-order polynomials on these three features in the regression. The higher order form merely improves the regression result to  ~70%. No second-order term has a significant coefficient in the model. Thus, although the simple linear regression model is learned through a greedy approach, we expect it's stable enough to indicate the importance of these three features in controlling the performance.

We also tried the transformations of the power family on the dependent variable *Y *using the Box-Cox method [11]. A lambda value other than 1 improves the  to about 90%. The three features mentioned above again show significance in the model. But some other features – the fraction of null sequences and density particularly – which are skipped in the first model show impact here. This confirms that the three features are likely important for affecting the performance, but we can't rule out other features.

It's no surprise that the sequence conservation (relative entropy) is key to the hardness of a dataset. It turns out that tools are actually quite robust with respect to the size of the dataset in a large range (up to 10,000 bp). Rather, the length of each single sequence has a bigger impact. This is somewhat supported by our discussion of the objective functions that sequences in a dataset should be considered as individuals. Also, it is connected to the position distribution information, as the longer each single sequence is, the more significant it becomes that the binding sites are not uniformly distributed in the sequences.

## 3 Can other information help?

The result of the multiple regression suggests a type of information that may help capture the hidden information in the motif's binding sites: the conservation of the binding sites' positions in the promoter sequences. It has been discussed in previous work (see [12]), but never integrated into the objective functions by the commonly used tools.

**Figure 6:**
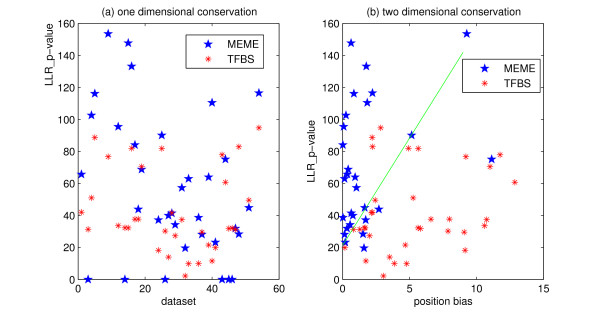
Position conservation information helps classification. In both figures, the *y*-axis is the negative log *p-*value of the log likelihood ratio of a motif in a dataset. The *x*-axis in (a) is the dataset index, in (b) it is the *p*-value of the Kolmogorov-Smirnov test on positions of a motif's binding sites, assuming a uniform distribution. Each point represents one dataset. For the same reason as in Figure 1, no "Real" type datasets are included. In (b) the straight green line decently classifies the two sets of points.

As discussed above, log likelihood ratio alone is unlikely to distinguish the true binding sites from the background noise. Figure 6(a) shows a different view of Figure 1. The (inaccurate) predictions from MEME serve not only as false positives versus the planted motifs, but also perhaps the hardest to separate from the true binding sites. A simple horizontal line classifier obviously can not separate the true binding sites from the predictions. In Figure 6(b), we introduce a second number in each dataset: we performed a Kolmogorov-Smirnov test on the positions of the binding sites, and calculate its *p*-value assuming a uniform distribution as the background model. Now on the 2*D *plane, the axes correspond to the motifs' conservation in both sequence and position. It's easy to see that even a straight line classifier *y - ax - b = *0 will separate the two sets decently. Let *Pr*_*llr *_be the *y *value, the negative log *p*-value of the log likelihood ratio, *Pr*_*pos *_be the *x *value, the negative log *p*-value of Kolmogorov-Smirnov test as explained above. Most true binding sites will fit *aPr*_*pos*_*- Pr*_*llr*_*+ b >*0, and most false predictions of MEME will fit *aPr*_*pos*_*- Pr*_*llr*_*+ b *< 0. The straight line in Figure 6(b) has parameters *a *= 13.5, *b *= 21.

This interesting result suggests a new form of objective function

**Figure 7:**
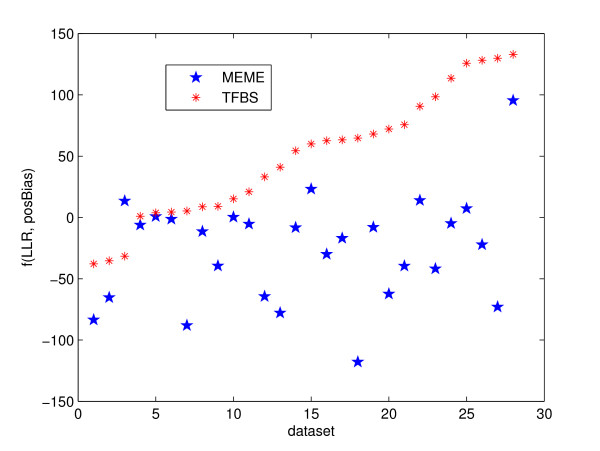
A new objective function using position information. The figure shows the same test as in Figure 1 on a new objective function. The x-axis tells the indices of the datasets, the y-axis the value of the objective function for the motif, either planted (red points) or predicted by MEME (blue points). Only datasets of "Generic" and "Markov" types are tested. For all but one of the datasets, the planted motif has a higher score than MEME's prediction.

*aPr*_*pos *_- *Pr*_*llr*_

against MEME's predictions for the value of a calculated from Figure 6(b). Figure 7 displays a very promising result, as for all but one of the datasets the planted motif has a higher score than MEME's prediction. Of course, this comparison is somewhat unfair to MEME, as it wasn't trying to optimize this function. But we can't help but ask this question: if we optimize this form of objective function, will we be able to improve on the predictive accuracy of MEME and other tools? The idea is very tempting, at least. Of course, the "new" pursued objective function may be some other function of these two types of conservation information, as it's not necessarily linear, or if it is linear, the coefficients *a *and *b *can vary from data set to data set.
